# Cap-Independent mRNA Translation in Germ Cells

**DOI:** 10.3390/ijms20010173

**Published:** 2019-01-05

**Authors:** Brett D. Keiper

**Affiliations:** Department of Biochemistry and Molecular Biology, Brody School of Medicine at East Carolina University, Greenville, NC 27834, USA; keiperb@ecu.edu; Tel.: +1-252-744-2656

**Keywords:** protein synthesis, eIF4 factors, RNA-binding proteins, maternal/paternal mRNAs, meiosis, gametogenesis, apoptosis, caspase, picornavirus

## Abstract

Cellular mRNAs in plants and animals have a 5′-cap structure that is accepted as the recognition point to initiate translation by ribosomes. Consequently, it was long assumed that the translation initiation apparatus was built solely for a cap-dependent (CD) mechanism. Exceptions that emerged invoke structural damage (proteolytic cleavage) to eukaryotic initiation factor 4 (eIF4) factors that disable cap recognition. The residual eIF4 complex is thought to be crippled, but capable of cap-independent (CI) translation to recruit viral or death-associated mRNAs begrudgingly when cells are in great distress. However, situations where CI translation coexists with CD translation are now known. In such cases, CI translation is still a minor mechanism in the major background of CD synthesis. In this review, I propose that germ cells do not fit this mold. Using observations from various animal models of oogenesis and spermatogenesis, I suggest that CI translation is a robust partner to CD translation to carry out the translational control that is so prevalent in germ cell development. Evidence suggests that CI translation provides surveillance of germ cell homeostasis, while CD translation governs the regulated protein synthesis that ushers these meiotic cells through the remarkable steps in sperm/oocyte differentiation.

## 1. Introduction

Translation initiation independent of the mRNA cap recognition is a mechanism now broadly accepted in mammalian cells [1]. However, when it was discovered over three decades ago, only invasive RNA viruses were thought to use this unusual mechanism to recruit ribosomes [2]. Picornaviruses in particular encode internal ribosome entry sites (IRESes) that act much like prokaryotic Shine Dalgarno sequences to provide viral RNAs an advantage over cellular mRNAs [3]. They exercise that advantage often by cleaving the eIF4G subunit of the host cell’s mRNA cap-binding complex [4,5]. In characterizing virus-infected cells, it was found that selected cellular mRNAs could also use cap-independent (CI) initiation, but these were thought to be anomalies. Cap-dependent (CD) initiation was presumed to suffice for “normal” cellular mRNAs, all of which had received a 7-methylguanosine (m7G) cap structure co-transcriptionally in the nucleus before export [6,7,8,9]. It is now recognized that the cellular context in which CI initiation occurs is not limited to virus-infected cells [1,2,10,11,12,13]. There are other contexts in which CI translation becomes a substantial protein synthetic mechanism in cells, notably apoptosis. For that reason, CD and CI translation mechanisms were viewed as separate traits of “happy” vs. “suicidal” cells, respectively [1,14,15,16]. Apoptosis unleashes endogenous caspase-3 that specifically cleaves eIF4GI and eIF4GII to render translation CI [13,17,18,19,20,21]. As a result, growth/differentiation-promoting CD translation is lost, and the remaining CI activity favors apoptotic mRNAs like Apaf-1, Bcl-2 and XIAP [20,22]. Evidence now suggests that CD and CI translation naturally co-exist in essentially all eukaryotic cells [23,24]. We do not yet know if germ cells invoke a regional distribution of CD vs. CI synthesis, but a balance between them may guide physiological processes required for attaining proper cell fates. Developmental apoptosis (often called “physiological apoptosis”) is one such process. It plays a vital role in sculpting embryonic cell lineages, maturing reproductive organs, and the homeostatic survival of the germline itself (reviewed in [25,26]).

Quite a number of cellular mRNAs, which are m7G-capped for stability, translate in a largely cap-independent fashion when the conditions present themselves [27]. Such mRNAs remain actively translating on polyribosomes in poliovirus-infected cells [28]. The identities of endogenous mRNAs that can use the CI translation mechanism also indicate CI translation has broader use than cellular crises. Some cellular mRNAs have demonstrable IRESes and encode proteins like the chaperone BiP [29,30], growth hormone FGF-2 [31,32], angiogenic factor VEGF [31,33], proto-oncogene c-Myc [34], pro-apoptotic Apaf-1 [35,36], anti-apoptotic Bcl-2 [37]. As more cellular CI mRNAs are discovered, it is becoming clear that many have functions unrelated to apoptosis or viral infection [38]. Their involvement in growth, signaling or stress-recovery suggests that cells “play both sides” of the cell death game using CI translation [13,16,23,29,32,38,39,40,41]. An ongoing controversy surrounds whether all these mRNAs contain canonical IRES elements because their initiation potential is not as strong as their viral counterparts [27,42]. More curious are the mRNAs with short 5′ untranslated regions (UTRs) or unusual 3′ sequence elements called cap-independent translational enhancers (CITEs) that do not undergo internal ribosome entry, but also derived no benefit from mRNA cap recognition [14,43,44,45]. These are truly “cap-independent” (CI), but their initiation elements contain no recognizable IRES sequence. Often their CI translation is not based on RNA sequence at all, but rather nucleoside base modifications. By way of example, hsp-70 mRNA uses 6-methyladenosine (m6A) residues to promote initiation by direct binding to eIF3 [46]. Since eIF3 is preloaded on 40S subunits, m6A-mediated initiation likely bypasses eIF4E and eIF4G altogether. Other modes of CI translation also do not require the IRES trans-acting factors (ITAFs) in the initiation complex that viral and apoptotic IRESes generally require [47,48]. It now seems clear that multiple routes to the ribosome are being discovered. The identities of mRNAs facile with each molecular mechanism are being compiled. But sorting out the identities and elements of CD and CI mRNAs does not get us closer to understanding how cells actually use the various modes of initiating translation.

**Figure 1:**
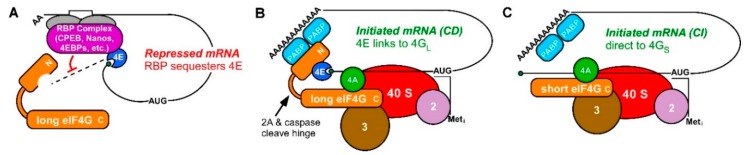
mRNA translational repression and CD and CI translation initiation complexes (**A**) RBP complexes bind sequence recognition motifs (white box) in the 3′ UTR mRNAs and often include an eIF4E-binding protein (4EBP). Protein-protein interactions within 4EBP-eIF4E-mRNA form stable mRNP complexes (purple) that inhibit the recruitment of eIF4E-bound mRNA to eIF4G (orange) and the ribosome. (**B**) Model of cap-dependent (CD) translation initiation utilizing the cap-binding protein eIF4E (blue). CD mRNAs are recruited to the 40S ribosomal subunit by binding of eIF4E to eIF4G and PABP. (**C**) Model of cap-independent (CI) translation. A short or cleaved form of eIF4G lacking an eIF4E-binding domain is still capable of recruiting mRNA to the 40S subunit by forming a CI complex with other eIFs directly on the mRNA.

Some hope may lay in the potential to characterize the CD and CI translation initiation complexes themselves. We now recognize that all eukaryotic cells make at least two forms of eukaryotic initiation factor 4G (eIF4G), one that recognizes the cap through eIF4E and another that circumvents cap-recognition and binds mRNAs directly [20,34,49,50]. Mammal genomes encode two long isoform genes (4G_L_: eIF4GI and eIF4GII), and one short form (4G_S_: p97/DAP5/NAT1); the latter lacks the binding site for eIF4E [20,34]. Cells artificially depleted of CD complexes (by disrupting 4G_L_) still initiate some portion of cellular protein synthesis [28,51,52,53]. The short eIF4G assembled into a CI complex is competent to recruit ribosomes to mRNAs, whether capped or uncapped [4,5,34,54]. Despite some remaining murkiness regarding which mRNAs use CI initiation and which use CD initiation [27], the availability of core complexes catalyzing both CD and CI initiation activities in somatic cells is widely accepted (Figure 1) [55,56]. But do these complexes have consequences in development?

## 2. Sorting Roles for CD and CI Translation in Differentiating Meiotic Cells

### 2.1. Translational Control in Development Has Focused on Repression

More than other cell types, germ cells (and embryos they produce) regulate the synthesis of new proteins (and thus cell fate) by mRNA translational control. Best described is the regulation by specific RNA-binding proteins (RBPs) that exert translational repression at the 3′ untranslated region (3′ UTR) and/or changes in mRNA poly(A) length [57,58,59]. However, mRNA regulation cannot be fully understood by repression alone [60]. Translation factors eIF4E and eIF4G must therefore work in concert with RBPs to regulate de-repression and *de novo* recruitment of ribosomes (Figure 1). The mechanisms of positive translational control in development remain poorly understood, though recruitment is arguably the important step in getting a protein made.

Unlike somatic cells that are susceptible to RNA viruses, germ cells have few endemic pathogens that might disrupt translation mechanisms. Thus, there was never a reason to question the prevalence of CD translation in these unusual cells. Yet, germ cells are known to use robust mRNA translational control to modulate gene expression. There is a prominent role for both mRNA poly(A) tail length and m7G cap-recognition in both the repression and activation mechanisms on controlled mRNAs [61,62,63]. One well-studied mechanism involves mRNAs repressed via a 3′ UTR-bound RBP (e.g., CPEB) that also sequesters eIF4E from eIF4G (Figure 1A). Elegant studies link the repressed CPEB-eIF4E mRNP to its hormone-induced activation. The recruitment involves coincident dissolution of the sequestered complex, cytoplasmic poly(A) elongation, and enhancement of eIF4E-eIF4G-PABP interactions to bring bound mRNAs to ribosomes [61]. Inverse regulation of ribosomal protein mRNAs occurs in the same cells upon their deadenylation [64,65]. Together these findings cement the notion previously demonstrated in vitro that mRNA caps and poly(A) tails act synergistically in translational control [66]. eIF4G coordinates eIF4E and PABP to promote the assembly of a “closed loop” circular mRNP that initiates translation (Figure 1B) [67]. Circularization also facilitates the recycling and re-initiation of post-termination ribosomes via ABCE1, thus increasing the mRNA’s translational efficiency [55,56,68]. Based on mounting examples of 3′ UTR-bound translational repressors in development, it seemed for a time that mRNP release, caps and poly(A) tails might tell us all we needed to know about translation in germ cells [61,63,69,70].

### 2.2. Germ Cell Translation Does Not Follow the Rules; the Prevalence of CI Translation in Frog Oocytes

**Figure 2:**
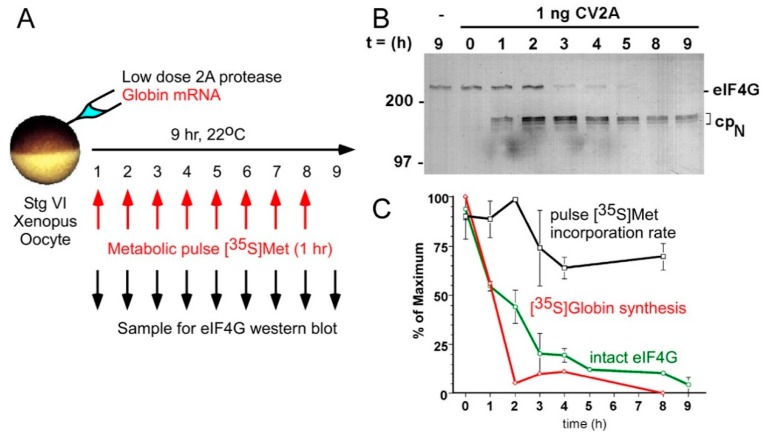
Progressive cleavage of *Xenopus* oocyte eIF4G in vivo efficiently blocks CD translation but allows 70% of protein synthesis to continue as CI translation. (**A**) A schematic of the experimental design. A suboptimal dose of recombinant coxsackievirus protease 2A (CV2A) was co-injected with purified beta globin mRNA. Oocyte eIF4G was cleaved progressively over 9 h, and 1 h pulses of [^35^S]methionine were administered for metabolic labeling of globin synthesis and total residual protein synthesis at each timepoint. (**B**) Western blotting for eIF4G1 to follow progressive cleavage to disrupt the CD form and produce the CI-only form (cpN) (**C**) Diminished beta globin translation (CD translation; red) correlates closely with eIF4G cleavage (green), while the protein synthetic rate (black) is largely refractory to loss of both. Co-injection of edeine (inhibits initiation events) suppressed 90% of synthesis (not shown), demonstrating that CI synthesis represents new initiation events. (Reproduced from Keiper and Rhoads, Nucl Acids Res [75] with permission from Oxford University Press).

In an effort to study the significance of CD translation and the m7G mRNA cap in vivo, we and other labs employed a very versatile germ cell, the meiotically arrested stage VI oocyte from the frog, *Xenopus laevis* (Figure 2) [71]. Isolated oocytes are as robust as rabbit reticulocyte lysates for protein synthesis, and can sustain translation initiation over a much longer time [72,73]. But unlike the reticulocyte, oocytes are largely resistant to competitive inhibition by the cap analog m7GTP [74]. To address the possibility that vertebrate oocytes have substantial CI activity, we assayed how much of endogenous mRNA translation was resistant to eIF4G cleavage by Coxsackievirus 2A protease [75]. This picornaviral protease specifically cleaves the hinge region of both eIF4GI and eIF4GII (4G_L_), as well as PABP, and abolishes CD translation [5,49,76,77]. Almost 70% of synthesis from ongoing initiation events remains active over hours, despite complete cleavage of eIF4G (Figure 2B). Removal of the cap-associated N-terminal domain (“cpN”, Figure 2) produces a residual eIF4G “core” (like 4G_S_) that no longer associates with eIF4E and the mRNA cap, but still faithfully assembles an initiation complex and recruits ribosomes to CI mRNA [78]. In the “CI-induced” oocytes, most endogenous housekeeping mRNAs, including actin, translate unabatedly for hours, sustained by demonstrable re-initiation events [75]. Globin mRNA (highly cap-dependent) injected into the same oocytes, loses its translational capacity in direct correlation with the loss of 4G_L_ (Figure 2B). This provided an interesting opportunity to address the developmental translational control event described above that occurs at oocyte meiotic maturation. Do the regulated mRNAs become recruited to ribosomes upon cytoplasmic poly(A) elongation in response to meiotic cell cycle progression (G2/M) [79,80] use CD or CI initiation? The subsequent study showed that intact 4G_L_ (and hence, CD initiation) is essential for entry of these cell-cycle regulated mRNAs into polyribosomes [81]. Cleavage of oocyte 4G_L_ prevents the translational recruitment of *c-mos* and cyclin B1 mRNAs, even though their poly(A) tails become elongated (even hyper-adenylated). Furthermore, the meiotic cell cycle arrest caused by abolishing CD initiation is not due to inhibition of protein synthesis *per se* because co-injection of an MPF extract (crude cyclin B/CDK2) restores cell cycle progression [81]. Conversely, others observed that intact oocyte eIF4G efficiently recruits IRES-containing mRNAs like EMCV and poliovirus transcripts, demonstrating that CD and CI initiation activities coexist in these germ cells (and presumably those of all species with like translational control) [82,83]. Importantly, EMCV mRNA is not enhanced by cleavage of 4G_L_, whereas polio mRNA is enhanced by 4G_L_ cleavage and mammalian ITAFs. Thus, sequence specificity also exists for CI mRNA translation in germ cells. Clearly there is a breadth of CD and CI activities that points to separate protein synthetic roles. Germ cells appear to maintain versatility in mRNA recruitment mechanisms to prepare them for the differentiation challenges ahead.

It was recognized early that both 4G_L_ (cap-associating) and N-terminally truncated 4G_L_ (non-cap-associating) can catalyze CI translation, and that the latter mimics 4G_S_ [78]. Naturally occurring 4G_L_ and 4G_S_ forms are found in all multicellular eukaryotes [3,56,84,85,86]. Oocytes are large cells arrested in the cell cycle, and much of their preparation for protein synthesis hints at the cell’s future. They position mRNAs and translation apparatus in place for the rapid differentiation of early blastomeres in the cleaving embryo [87]. Substantial CI translation has been observed in germ cells/embryos in addition to frogs; animals as diverse as worms, flies, and humans [23,50,75,88,89], and even in plants [41]. The universality of germline CI translation suggests it is an integral theme programmed into this specialized, immortal cell lineage; germline. We postulate that broad translational versatility allows a subset of native germ cell mRNAs (both regulated and house-keeping) to utilize a mode of CI, and perhaps even IRES-mediated, translation. Importantly, such mRNAs are regulated by recruitment events that are distinct from CD mRNAs, which rely on eIF4E-linked repression/de-repression and poly(A) elongation [61,81,90,91,92,93,94,95]. By coordinating 3′ UTR repression (see Section 2.1), CD translation, and CI translation separately, an egg may set up localized synthesis of proteins for the subsequent differentiation steps in early embryo blastomeres [87].

### 2.3. A Use for CI Translation in Germ Cell Homeostasis; Evidence from Worm Oocytes

**Figure 3:**
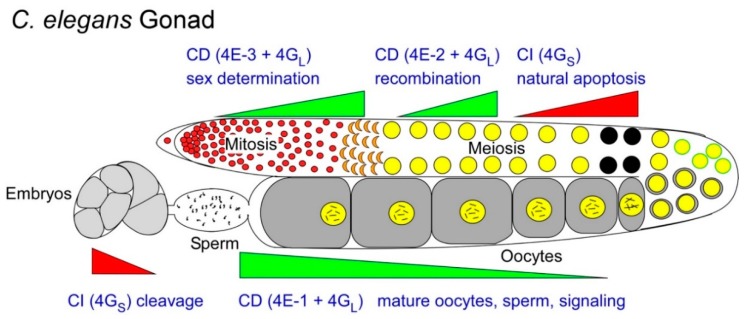
Alternate or overlapping use of CD (green wedges) and CI (red wedges) translation mechanisms in developing *C. elegans* oocytes and sperm. The wedges represent predominant, rather than exclusive translation mechanisms used in each germ cell transition, and are approximated from observed mutant and RNAi phenotypes of eIF4E (-1, -2 and -3) and eIF4G isoforms. A series of anecdotal studies from various animal germ cell models suggest that germ cells alter the balance of CD:CI translation at various times, or in various populations, to promote differentiation fates (e.g., maturation, sex-determination, apoptosis, etc.; see Section 2.1, Section 2.2, Section 2.3 and Section 2.4) The balance is held by active fractions of eIF4G_L_ vs. eIF4G_S_. Within the CD translation mode, 4G_L_ has multiple options of eIF4E forms to bind, and each specifies a further select subset of mRNAs to recruit (Section 2.5). 4G_S_ presumably recruits CI mRNAs directly. Black circles indicate a subset of germ cells selected for physiological apoptosis.

In the nematode worm *C. elegans*, the pathways for apoptosis [96], germ cell development [97] and mRNA translational control [58,69] are each well understood. The confluence of these three fields has been highlighted to explore the control of apoptosis in gamete development. What has been learned is that apoptotic mechanisms are used as modes of homeostatic control. These mechanisms govern which germ cells survive as well as provide a means to mature the surviving gametes. Apoptosis balances mitotic proliferation with the biosynthetic capacity of the gonad to produce potent and viable gametes [26,98,99]. Physiological germ cell apoptosis in worms removes nearly half of cell population during oogenesis (Figure 3; [98]). In so doing, dying cells provide their sibling germ cells with cellular components for survival, functionally similar to nurse cells from insect germaria and Sertoli or cumulus/granulosa cells in the vertebrate testis or ovary, respectively [25].

Not unexpectedly, mRNA translational control is a contributor to apoptosis which ensures homeostatic balance of the germ cell population. The activities of numerous RBPs are essential to stave off germ cell apoptosis. Some are involved in mRNA repression, like CPEB and PUF-8, or resident in mRNA storehouses, like P granule proteins PGL-1 and PGL-3 [100,101,102,103]. Still others play roles in mRNA stability like the helicase complex CGH-1/CAR-1, which localizes to both P granules and P bodies [104,105,106]. Even the translation initiation factors themselves are intricately involved in the germ cell decision to survive rather than apoptose. Our studies point to a balance of CD and CI mechanisms to balance survival with cell death. Here the utilization of both short and long native isoforms of eIF4G is again at work. Interestingly, *C. elegans* encodes just one eIF4G gene, *ifg-1,* from which to make 4G_L_ and 4G_S_. We discovered that worms transcribe two major mRNAs from the *ifg-1* gene that independently encode a p170 isoform (4G_L_) that contains the N-terminal domain that binds each of five worm eIF4Es (IFEs 1-5), and a p130 (4G_S_) that lacks the eIF4E-binding domain [84]. Selective depletion of 4G_L_ by RNAi against p170 mRNA induces CI conditions and dramatically expands germ cell apoptosis with little effect on somatic growth [84]. Apoptotic cells assemble Apaf-1 (CED-4) into apoptosomes and the doomed oocytes are engulfed by the gonad sheath, exactly like native events (Figure 3). More importantly, the translational efficiency of the Bcl-2 and BiP mRNAs is modestly enhanced under 4G_L_-depleted conditions in vivo, while that of most other mRNAs was reduced [23].

By contrast, inhibition of all translation initiation would be expected to prevent both growth and development. Indeed, depletion of both 4G_S_ and 4G_L_ by RNAi stunts somatic growth and arrests larval development, precisely mimicking the arrest observed in an *ifg-1* null mutant strain [84]. The disparity of these RNAi phenotypes made clear that a CD:CI imbalance, and not diminished protein synthesis *per se*, induces germ cell apoptosis. In situ metabolic labeling showed equivalent total protein synthesis activity in control and 4G_L_-depleted gonads that was uniform across surviving and apoptosing sibling germ cells (unpublished data). This observation, too, may suggest that neither the CD nor CI mechanism limits overall protein synthesis, but each works instead with subsets of mRNAs. Moreover, mutations in the worm Apaf-1 (*ced-4*) or caspase (*ced-3*) genes fully blocked the CI-induced germ cell apoptosis [89]. Therefore, the balance of CD:CI translation acts as an upstream signal in the canonical cell death pathway rather than a downstream consequence of the dying process. The pathway also incorporates a means of positive feedback for apoptosis, once begun. The CED-3 caspase was shown to proteolytically cleave 4G_L_ (p170) in vitro and in vivo [89] in identical fashion to mammalian eIF4G [17,18,19]. The implication is that selected germ cells respond to unbalanced CD:CI translation in a way that changes their survival capacity relative to their siblings (Figure 3). Since *C. elegans* germ cells share cytoplasm through syncytial connections, it remains to be determined how CI translation is locally maintained in only those cells destined to die (black circles). However, this must also be the case for other translational control events that act locally at mitotic and meiotic transitions in early germ cell development [58].

The balance between survival and apoptosis is also linked to the MAPK pathway [100,107], which is coincidentally also important for mRNA translational de-repression [69]. mRNA repression by RBPs like Nanos must be maintained for oocyte survival. Disruption of Nanos binding to mRNA targets also expands germ cell apoptosis, and likewise requires signaling through Apaf-1 [102]. Nanos regulation was first discovered in fruit flies [70,108,109] and is also observed in vertebrates [110]. Unlike the regulation by CPEB described in Section 2.1, Nanos and Pumillio regulate mRNAs independently of poly(A) modifications [111]. mRNA regulation by Nanos can occur via CD translation [110] or via CI translation, if the mRNAs contains an IRES [88]. Thus, depending on the type of mRNA repressed by a 3′ UTR-binding RBP, it is possible for either CD or CI initiation mechanisms to mediate their positive activation. The variety of both negative and positive modes represent layers of potential regulation options and challenge our fundamental understanding of mRNA translation initiation as it occurs in germ cells.

### 2.4. Germ Cells also Use CI Translation of “Death-Promoting” mRNAs to Differentiate

Given what is known about CI translational control, the underlying mechanism appears to be available, but not prominent, in somatic cells. We suggest that CI translation activity is more pronounced in germ cells. Maintaining a CD:CI balance that is closer to some critical threshold allows germ cells to use the “cell death”-like tendencies in two ways. The first is to govern homeostatic selection within the germ cell population to survive overt physiological apoptosis. The second is perhaps less obvious. There is significant use of apoptotic-like activities in spermatogenesis and oogenesis that are required, not for cell death, but for their normal maturation to gametes. The exercise of “apoptotic-like” destructive activities are critical for maturation of the surviving germ cells that differentiate. Spermatogenesis takes particularly broad advantage of these intracellular “culling” activities without killing the host cell.

Non-lethal use of death-promoting proteins is vital for reproductive strategies in many species. As anecdotal examples, apoptosis-unrelated roles for endonuclease G, Bcl-X and its family members, and numerous caspase-associated proteins are part of adaptive responses that take place during the dramatic differentiation of gametes [112]. Expression of multiple caspases and the FADD death-receptor is essential for *Drosophila* spermatogenesis [113]. Apaf-1 protein is also highly expressed in spermatogenesis and is required for cell individualization in worms, flies and mice [114,115,116]. In somatic cells Apaf-1 functions in a non-apoptotic role in the DNA damage checkpoint [117], so it may similarly oversee the correct resolution of meiotic recombination and DNA crossovers in germ cells. The mRNA translational control observed during apoptosis may have corresponding non-lethal uses in gamete maturation. In addition to eIF4G isoform switching (described in Section 2.5 below), other translation-related factors aid germ cells and embryos in sustaining appreciable CI activity. Y-box proteins are abundant RBPs in germ cells that stabilize mRNPs required later for oocyte and sperm viability [118]. Interestingly, maternal Y-box proteins that persist in embryos following fertilization have been shown to enhance the CI translation of snail mRNA [119]. Snail has important roles in subsequent mouse epithelial differentiation. For reasons yet unknown, the translational apparatus is set up to allow substantial flexibility between CD and CI translation along the germ cell to gastrula axis.

### 2.5. Even CD Translation in Germ Cells Comes in Multiple Flavors

Both eIF4E and eIF4G, which cooperate in CD translation, have been implicated in various cell fate decisions and even oncogenesis [120,121,122,123]. Animal and plant studies show that germ cell and embryonic fates are greatly affected by the eIF4 factor complexes unique to those cells [41,60,62,92,94,114,124,125,126,127,128,129,130,131,132,133,134]. Factors eIF4E is highly conserved across species (yeast to human) and is universally represented by multiple isoforms. For example, three eIF4E proteins have been characterized in mammals, five in *C. elegans*, three in *Xenopus*, three in plants, three in zebrafish, and eight in *Drosophila* [92,130,133,135,136,137,138]. Germ cells in these species express multiple eIF4Es that are unique to, or predominate in, only those cells.

The employment of genetics with biochemistry has helped to identify unique roles for eIF4E and eIF4G isoforms in reproduction. Both fly and mouse spermatocytes express sperm-specific eIF4Gs that have specialized roles [85,86,124,139]. The eIF4G homologues Off-schedule (eIF4G2) and Repro8 (eIF4G3) are regulators of meiotic progression and differentiation. *Drosophila* spermatocytes depleted of eIF4G2 show growth defects and accumulate the CDK inhibitor protein, RUX, likely as a growth checkpoint before meiotic division [139]. Mouse spermatocytes lacking eIF4G3 arrest in meiotic prophase and are unable to translate Hspa2 mRNA, which is necessary for activation of meiotic prophase kinase CDC2A [86]. Germ cell eIF4Es in plants, flies and frogs also have unique roles in development judging by the reproductive phenotypes resulting from their deficiencies [126,133,135,138]. In a few cases, specific mRNAs have been shown to respond to a specific eIF4E isoform. A unique *Drosophila* eIF4E regulates the translation of oskar mRNA, which is necessary for embryonic posterior patterning [94,140]. *Xenopus* eIF4E1b was identified in an mRNP complex responsible for the suppression of meiotic maturation in early stage oocytes [92]. This eIF4E1b associates with a novel 4EBP called eIF4E-T that transports and sequesters mRNPs as a form of repression. eIF4E-T is itself a determinant of germ cell differentiation [141], further linking CD regulation to cell fates (Figure 3).

**Figure 4:**
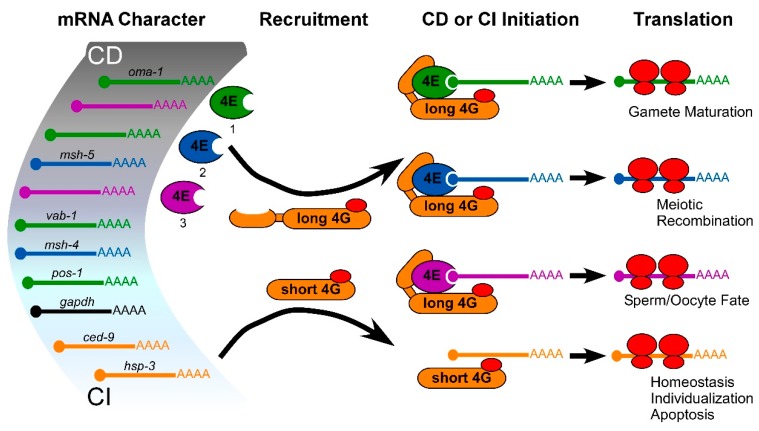
Model of selective, positive translational mRNA recruitment in germ cells that is based on eIF4E and eIF4G isoforms represented in *C. elegans*. Multiple studies suggest that eIF4E and eIF4G isoforms are selective in translating mRNAs (see text), and there may be a gradation of cap-dependent (CD) to cap-independent (CI) requirements to the selection. Identified CD mRNAs recruited to ribosomes by eIF4E-1 (green), eIF4E-2 (blue) or eIF4E-3 (purple) via association with long eIF4G (yellow; 4G_L_) in germ cells are shown [60]. Short eIF4G isoform (4G_S_) recruits identified CI mRNAs without eIF4Es [23].

The utility of multiple eIF4E isoforms is most evident in *C. elegans* development. Worms express five non-redundant isoforms from independent genes (eIF4Es are called IFE-1 to -5). At least three forms function in the germ line, and one exclusively in somatic cells [130]. Studies using strains with null mutations in individual eIF4E genes show that each deficiency exhibits a different developmental phenotype, and most reduce fertility [114,129,131,142,143]. Polysome and reporter analyses show that each isoform preferentially recruits a unique subset of mRNAs for CD translation (Figure 4) [114,131,144,145]. Germline eIF4E-1 (IFE-1), for instance, is the key translational regulator of late sperm and oocyte progression [114]. Without it, secondary spermatocytes are unable to complete cytokinesis, late oocytes grow and mature poorly, and any fertilizations culminate in embryonic arrest. Polysome bioinformatics show that eIF4E-1 recruits critical mRNAs (*mex-1, oma-1, glp-1, gld-1, pos-1, pal-1, vab-1, rab-7*, etc.), many of which encode proteins used for meiotic maturation or embryo differentiation [114,144]. Loss of isoform eIF4E-2, on the other hand, causes oocytes to be highly sensitive to meiotic catastrophes because of defects in chromosome repair [131]. As might be expected, eIF4E-2 recruits key mRNAs (*msh-4, msh-5*) for meiotic chromosome segregation. Perhaps not surprisingly, eIF4E-1 and -2 localize differently in growing oocytes. eIF4E-1 joins large mRNPs (P granules) by binding to PGL-1, whereas eIF4E-2 is diffuse in oocyte cytoplasm [129,131]. eIF4E-3 is perhaps the least understood isoform, and its mRNA targets are as yet unknown. eIF4E-3 promotes the decision between the female and male gamete cell fates, and associates with yet another mRNP, the OMA granule [141,143,146]. Non-regulated mRNAs (like beta-tubulin or GAPDH) appear to be indiscriminate in their choice of eIF4E isotype [23,114]. We propose a model in which a divergent CD and CI translational apparatus carries out critically important mRNA selections that alter germ cell fates (Figure 4 and [60]).

### 2.6. Potential Relevance of CI Translation to RNA Viruses That Cause Birth Defects

The recent epidemic of Zika virus infections that cause fetal microcephaly brings to light a potential crossroad for CI translation in germ cells, susceptibility to RNA viruses, and downstream embryonic development. The pathology observed in Zika-induced birth defects is attributed to neural tube malformation late in embryogenesis, but there is evidence that the virus may infect/accompany sperm or eggs [147]. Zika is an RNA flavivirus that is transmitted to the conceptus by either a male or female infected parent [148]. Like many RNA viruses, it alters mRNA translation mechanisms to commandeer host protein synthesis [149]. Zika replication is inhibited by an eIF4A inhibitor, silvestrol, which points to a potential role for eIF4 dysregulation in its pathology [150]. Heterozygous mutation of eIF4A4 in mouse embryos results in neural defects similar to those found in Zika pregnancies [151]. Zika infection also induces the unfolded protein response (UPR) to disrupt host translation initiation via eIF2 phosphorylation [149,152]. Many RNA viruses use UPR in conjunction with CI translation to coopt host protein synthesis. These are largely anecdotal connections to mRNA initiation activities, but an understanding of the balance of CD and CI mRNA translation will be useful to determine how “ripe” germ cells are for Zika infections. 

## 3. Conclusions

There was previously little reason to consider roles for CD and CI initiation mechanisms in germ cells, which already possess a large repertoire of mRNA translational control events. And it is worth noting that the relevance of CI translation in vivo is still questioned [153]. But recent observations show a striking diversity of eIF4 factors expressed in germ cells [60,136,154], and mutational analyses has made it clear they have independent roles in developing cell types. Such observations strongly suggest that CD and CI mechanisms may be as diverse as the many RBP-mediated translational repression schemes already characterized in germ cells and embryos. Yet we know considerably less about positive translational control than we do about negative control in these cells. Given that translation factors (particularly eIF4Es) are more evolutionarily conserved than are RBPs, it stands to reason that the eIF4 complexes in germ cells will function as interchangeable modules. This suggests that alternative complexes form, isoforms become sequestered or localized, and relative affinities vary (e.g., eIF4E-eIF4G_L_-mRNA-RBP vs. eIF4G_S_-mRNA-RBP). But all such complexes will contribute in a combinatorial manner to mRNA recruitment potential for translation. Future studies to untangle these complexities in vivo will be facilitated by recent tools (i.e., CRISPR technology, fluorescent translational reporters, polysomal RNA bioinformatics, RiboSeq, and mRNP proteomics) together with versatile genetic animal models. We seem poised for molecular dissection of CD and CI translation to identify functional complexes (RNA and protein) and their roles in differentiation. This initiative bears some resemblance to emerging interest in CI vs. CD translation mechanisms in oncogenesis and cancer progression [155,156,157]. Germ cells/embryos and cancers have revealed over time many remarkable parallels in metabolism, cell cycle and growth, so parallels in translation mechanisms might also be expected.

## Abbreviations

UTR	Untranslated region
eIF	Eukaryotic (translation) initiation factor
4G_S_	Short form of eIF4G
4G_L_	Long form of eIF4G
PABP	Poly(A) binding protein
IRES	Internal ribosome entry site
RBP	RNA binding protein
EMCV	Encephalomyocarditis virus
mRNP	Messenger ribonuclear protein
CI	Cap-independent
CD	Cap-dependent
CITE	Cap-independent translational enhancer
CPEB	Cytoplasmic polyadenylation element binding protein
MPF	Maturation promoting factor (cyclin B/CDK2)
